# Distributed Sensing Based on Interferometry and Polarization Methods for Use in Fibre Infrastructure Protection

**DOI:** 10.3390/s19081810

**Published:** 2019-04-16

**Authors:** Petr Munster, Tomas Horvath, Josef Vojtech

**Affiliations:** 1 Department of Telecommunication, Brno University of Technology, Technicka 12, 616 00 Brno, Czech Republic; horvath@feec.vutbr.cz; 2 Department of Optical Networks, CESNET a.l.e., Zikova 4, 160 00 Prague, Czech Republic; vojtech@cesnet.cz

**Keywords:** distributed sensing, digging detection, fibre infrastructure protection, interferometer, polarization

## Abstract

Fibre optic infrastructures are very important, and therefore, it is necessary to protect them from fibre cuts. Most fibre cuts are caused by digging activity, and many network operators seek appropriate solutions enabling detection of possible unexpected events (predict these cuts) and subsequent network outages. In most cases, there is no need to locate events, and only information regarding the occurrence of the event is sufficient. Direct detection-based distributed fibre optic sensing systems appear to be an appropriate solution, allowing digging to be detected before the fibre breaks. The average power of such signals is relatively small, and there is no interference with other signals in the fibre. We performed laboratory measurements to compare the sensitivity and accuracy of interferometric and polarization systems for acoustic vibrations. In the case of interferometric systems, the reference and sensing arms were in the same cable, and both were subjected to acoustic vibrations.

## 1. Introduction

Fibre optic infrastructure is very important because it enables data transmissions not only in long-reach networks but also in metro and access networks [1]. Optical fibre is the only medium that can satisfy current and future requirements—large bandwidth, high speeds, low insertion losses, low costs, and the ability to allow signal multiplexing. Fibre optic infrastructure may be threatened by fibre cuts during construction work. An interruption of data transmission causes additional costs. For example, the Czech national research and education network (CESNET) experienced ≈120 outages between 2016 and 2017, and the number of outages caused by fibre cuts was ≥10%. Moreover, in cases of special networks such as the national research and education networks (NREN), not only data but also special services may be transmitted (e.g., accurate time, stable frequency or quantum key distribution). Protection of fibre infrastructure by a distributed sensing system may save not only costs but also time necessary for repairs, thereby increasing the quality of the network [2,3].

CESNET is the NREN in the Czech Republic, and in contrast to commercial network operators, it allows transmission of not only data but also special non-data services. Each service must have allocated bandwidth (part of spectrum in the fibre) that is reserved all along the photonic path. It is important to carry this path over a network with minimal impact. CESNET ensures transmission of data, accurate time, and stable frequency. New and rapidly growing services are distributed sensing a quantum key distribution (QKD). Distributed sensing can be used, for example, for monitoring of seismic activity, monitoring of digging activity along the fibres, or for monitoring events in fibre surroundings (train positions, aircraft landings, and others). QKD is a way to secure communication in a public network. The method uses cryptographic protocols and enables two end-users to establish a shared secret key for secure communication.

The main contribution of this article is a basic comparative measurement of two techniques used for distributed fibre optic sensing. Based on results from laboratory measurements, long-term measurement on a real telecommunication network will be prepared.

The structure of this paper follows. Section 1 provides an introduction to the topic. Section 2 deals with the basics of distributing sensing systems. In Section 3, we present a measurement setup description, and the results and discussion are presented in Section 4. Section 5 concludes the paper.

## 2. Distributed Fibre Optic Sensing

Distributed fibre optic sensing is a rapidly growing field for application of fibre optics, primarily because of the increasing availability of components and the falling prices of the main components [4]. This article compares two techniques that can be used to detect acoustic vibrations around fibres. These techniques meet the basic requirements of deployment in telecommunications: reasonable costs, low power levels and therefore minimal probability of interfering with other signals, i.e., simple signal processing. On the other hand, in basic configurations, localization of events is not possible (for localization, it is necessary to use two interferometers or to combine with another technique). There is also a technique enabling direct localization of events based on Rayleigh back-scattering [5]; however, this system uses high-power pulses that can interfere with data signals. Moreover, these systems are costly.

### 2.1. Standard Dual Fibre Optic Interferometers

**Figure 1:**
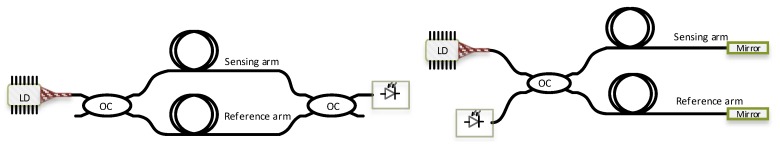
Mach-Zehnder interferometer (**left**), Michelson interferometer (**right**). LD: Laser diode, OC: Optical coupler.

Light interference can be observed for coherent sources of light radiation and the superposition of waves. Interferometers work on the principle of interference and are used for very accurate measurements [6]. Optical phase shifts as small as $10^{-6}$ radian can be detected [7]. Beams from the same source travel by two different paths—one path is a sensing arm, and the second is a reference arm (we consider only two beams, derived from the same source but traveling along two separate paths). Interferometers evaluate optical path differences between sensing and reference arms as a phase change [6]:

(1) $$\Delta \Phi =\frac{2\pi }{\lambda }\Delta x=k\cdot \Delta x$$

where λ is the wavelength and $\Delta x=x_{2}-x_{1}$ describes the optical path difference. Based on theory, the reference arm should be separated from the source of vibrations. However, we have verified by measurement that two fibres in the same optical cable can also be used. There are two main configurations of interferometers used for acoustic vibration detection—the Mach-Zehnder and the Michelson interferometer (there is also another configuration, the Sagnac interferometer, but for fibre infrastructure protection, it is not often used). As can be seen in Figure 1, both configurations are similar, and the only difference is that the Mach-Zehnder interferometer uses a double-ended setup and the Michelson interferometer is single-ended (and is more accurate as light passes through the fibre twice). Despite high sensitivity to many physical phenomena such as temperature or stain changes, the main advantages of interferometers are their very fast responses and good potential for multiplexing. Total measurement distance is given by a coherence length of a laser—optical path difference between two arms of interferometer must be less than or equal to the coherence length.

Several papers describing interferometers as distributed fibre optic sensing technologies have been presented. For example, the authors in [8] describe dynamic strain measured by Mach-Zehnder interferometric optical fibre sensors and signal demodulation using a 3 × 3 coupler. A distributed fibre vibration sensor utilizing dispersion induced walk-off effect in a unidirectional Mach-Zehnder interferometer was presented by Chen et al. [9]. The authors in [10] describe dual parameter fibre optic sensor combining a Fabry-Perot and a Mach-Zehnder interferometer. We have presented some basic results regarding to interferometry based sensing systems in [11]. The paper described comparative measurement of distributed sensing systems, but only the basic idea without more experimental measurement results was presented.

### 2.2. Fibre Optic Polarization Interferometers

A special group of interferometer sensors are polarization interferometers. The main advantage of these sensors is that the total range is not limited by the coherence length of a laser. Standard single-mode optical fibre supports two degenerate modes of orthogonal polarization. The polarization state of light in a single-mode optical fibre is very similar to that of a plane light wave in free space; it could be linear, elliptical or circular [12].

All the important features of a light wave follow from a detailed examination of the Maxwell equations. Electromagnetic waves have two polarizations along the *x* axis and along the *y* axis. The general form of polarized light wave propagating in the z direction can be derived from two linear polarized components in the x and y directions [13]:

(2) $$E_{x}\left(z,t\right)=E_{0x}\cos\tau_{\omega }+\phi_{x}$$

(3) $$E_{y}\left(z,t\right)=E_{0y}\cos\tau_{\omega }+\phi_{y}$$

$E_{0x}$ and $E_{0y}$ are maximum amplitudes of electric field, $\phi_{x}$ and $\phi_{y}$ are the phases, and $\tau_{\omega }=\omega t-kz$ is the so-called propagator and describes the propagation of the signal component in the z-direction [14].

From the equations above, we can obtain the polarization ellipse that presents some important parameters enabling the characterization of the state of light polarization (SOP) [13]. The light wave components in terms of complex quantities can be expressed by means of the Jones vector. Whereas the polarization state is influenced by the surrounding events, if we evaluate the polarization changes, then we can evaluate the events along the fibre (temperature, stain, vibrations). Using polarization for distributed sensing was described by [2,15] .

## 3. Experimental Setup

**Figure 2:**
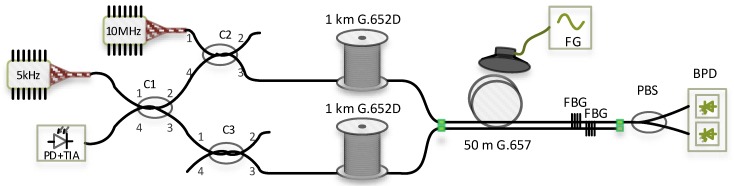
Experimental setup for simultaneous measurement of two sensing systems. FG: Function generator, PBS: Polarization beam splitter, FBG: Fibre Bragg grating, PD: Photodetector, TIA: Transimpedance amplifier, BPD: Balanced photodetector LD: Laser diode.

The experimental measurement scheme is shown in Figure 2. The scheme consists of two separate sensing systems in one setup. Using one complex setup for both measurements ensured almost identical conditions for both systems.

**Figure 3:**
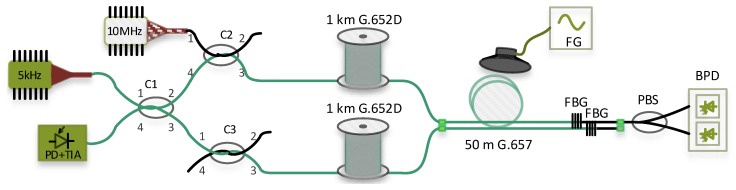
Experimental setup of the Michelson interferometer is highlighted in green (active components are also highlighted in green).

Ultra-stable and narrow linewidth laser source PureSpectrum™-NLL (less than 5 kHz, corresponding to approximately 19 km coherence length) with a wavelength 1550.92 nm is used for the Michelson interferometer setup. A continuous wave (CW) signal is in fused optical coupler C1 (2 × 2, 50:50 split ratio) divided into sensing arm and reference arm. Both signals then travel through couplers C2 and C3. While coupler C2 is used for launching a signal from the second laser into the fibre under test, coupler C3 has only a balancing function of the second arm: phase and insertion loss compensation. As mentioned above, an interferometer requires two arms with similar lengths (more precisely, the difference in the length of the arms must be less than the coherence length of the laser). For the measurement we used two separate 1 km long spools of optical fibre G.652D followed by 50 m long fibre to the home (FTTH) cable G.657A (four coloured fibres in jelly, aramid yarns, PE (polyethylene) outer sheath). As a mirrors Fibre bragg gratings (FBGs) with a reflectance about 90% were used. The Bragg wavelength corresponds to the central wavelength of the narrow linewidth laser source, and the bandwidth is less than 0.1 nm. Signals corresponding to the Bragg wavelength are reflected back to coupler C1, and other signals pass through. The reflected signals from both arms interfere in coupler C1. The final response is then detected on a photodetector connected to port 4 of coupler C1 with an integrated transimpedance amplifier. The experimental setup of the Michelson interferometer is shown in Figure 3 and is highlighted in green.

**Figure 4:**
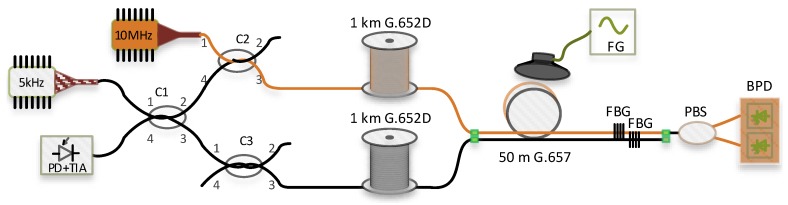
Experimental setup of polarization interferometer is highlighted in orange (active components are also highlighted in orange).

The polarization-based sensing system scheme is simpler, and only one fibre is needed (see Figure 4). A laser diode with a linewidth of approximately 10 MHz generates the CW signal that is launched through the coupler C2 into the fibre being tested. The laser wavelength is 1555 nm, which is different from the Bragg wavelength signal that is not reflected by FBG and passes to the polarization beam splitter that splits a single input into its orthogonal linear polarizations through two fibre outputs. As a receiver, we used a balanced detector. Two optical input signals are subtracted from each other, resulting in common mode noise cancellation. Using a balanced detector allows the extraction of small changes in the signal path from the interfering noise floor [16]. Although most papers describing the measurement of acoustic vibrations with polarization changes use a polarimeter (as in [2]) we decided to use a balanced photodetector (BPD). By using a BPD, we can directly evaluate frequency of acoustic vibration instead of angular changes. Moreover, a BPD is a less expensive solution than a polarimeter.

As a source of vibration, we used a loudspeaker and a signal generator. While both fibre spools were isolated from the source of acoustic vibration as much as possible (they were placed in another part of the room, laid on the foam to insulate the vibration from the floor), the cable was attached directly to the speaker with tape. In the case of the Michelson interferometer, both arms (sensing and reference) were in the same cable, and the reference arm was therefore not isolated from sources of vibration. Thanks to a slightly different position of both fibres in the cable, each fibre was affected by acoustic waves differently. Acoustic wave from a loudspeaker causes refractive index changes in the fibre, and light travels by a different path. Electrical signals from both detectors are acquired using a 100 kS/s (Samples per second) sampling rate for subsequent processing.

## 4. Results and Discussion

In real networks, fibres are in cables that are in high-density polyethylene (HDPE) conduits and are more than 70 cm underground, and HDPE filters out higher frequencies. That is why, for laboratory measurements, we chose frequencies of 1025 Hz, 530 Hz and 130 Hz. A harmonic signal was generated by an arbitrary waveform generator (AWG) and a loudspeaker.

We also tried to evaluate systems sensitivity on mechanical vibrations near the real fibre optic cable, but because the acoustic vibrations were not precisely defined, the results are more of a demonstration and function test.

### 4.1. Sine Wave, Frequency 1030 Hz

**Figure 5:**
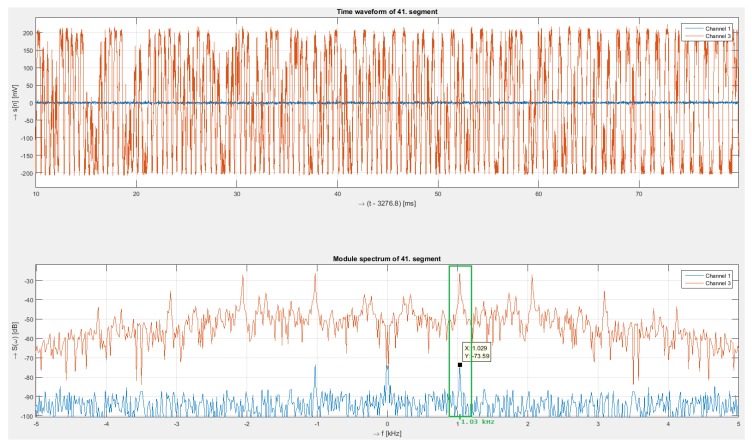
Received signals (**upper**) from both sensing systems (interferometer—red signal, polarization—blue signal) and their spectra (**below**) for 1030 Hz.

The highest test frequency of a harmonic signal was 1030 Hz with an intensity 10 $V_{pp}$ (peak-to-peak voltage). Figure 5 shows time signal and corresponding spectra of the interferometry system and the polarization system.

In a long-term test, the measured frequencies for both systems corresponded exactly to the generated frequency from the generator, as can be seen in Figure 5. From the time domain signal of the interferometry system, a modulated acoustic signal can be directly observed and signal intensity is relatively high, peak value is approximately –28 dB. The signal from the polarization system was weak, and in the time domain, it was not possible to evaluate the modulated signal, unlike the interferometric system. The signal peak value was approximately 46 dB lower, with a value of –74 dB. In the interferometry system, sensitivity was much higher; however, sub-harmonic frequencies can be seen in the spectrum of the signal.

**Figure 6:**
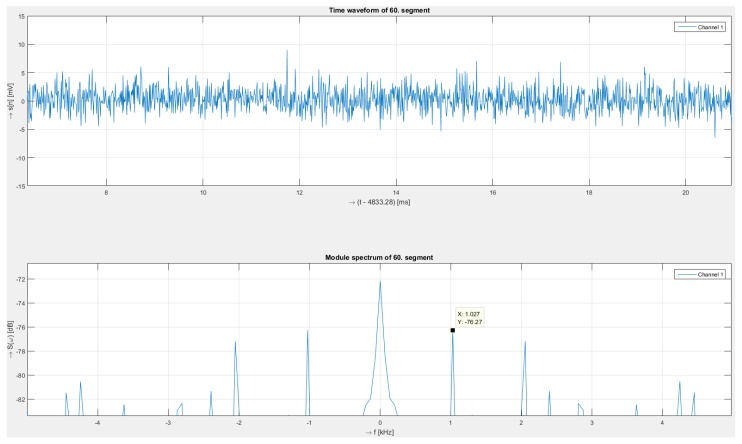
Detail of received signal (**upper**) for polarization measurement and corresponding spectra (**below**) for 1030 Hz.

Figure 6 shows the details of the response from the polarization system. In the time signal, the amplitude fluctuates, making it difficult to recognize the modulated signal, but the spectrum shows a peak at a frequency of approximately 1027 Hz and with an intensity of –76 dB. These values correspond to a short time window, so they are slightly different from long-time values, which are more averaged.

### 4.2. Sine Wave, Frequency 530 Hz

**Figure 7:**
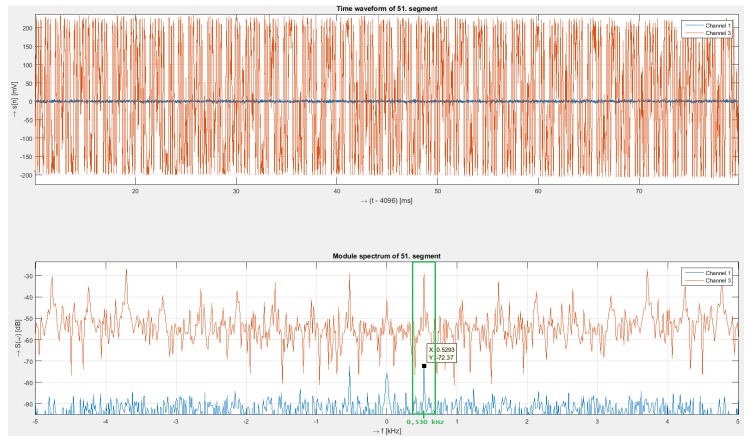
Received signals (**upper**) from both sensing systems (interferometer—red signal, polarization—blue signal) and their spectra (**below**) for 530 Hz.

Next, measurements were conducted for a harmonic signal with a frequency 530 Hz and an intensity of 10 $V_{pp}$. Figure 7 shows the time signal and corresponding spectra of the interferometry system and polarization system.

For long-term testing, we measured frequencies from both systems corresponding to the generated frequency 530 Hz from the generator, as can be seen in Figure 7. The time domain signal of the interferometry system captures the modulated frequency, while the signal from the polarization system is very weak. The peak values of the interferometry system were approximately –30 dB, and –72 dB for the polarization system.

In addition to the main frequency at 530 Hz, we can see in the spectrum of the interferometry system its multiple sub-harmonic frequencies with relatively high intensity.

### 4.3. Sine Wave, Frequency 130 Hz

**Figure 8:**
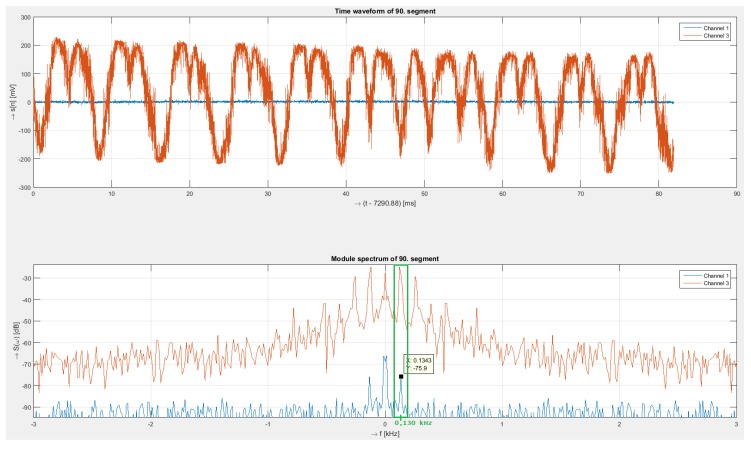
Received signals (**upper**) from both sensing systems (interferometer—red signal, polarization—blue signal) and their spectra (**below**) for 130 Hz.

The last measurement under laboratory conditions was performed for a harmonic signal with a frequency of 130 Hz and an amplitude of 10 $V_{pp}$. In Figure 8, we can see the time signal and corresponding spectra of the interferometry system and polarization system.

As in the previous cases, both systems measured the frequency correctly but with different intensities. While in the case of the interferometer, the signal was relatively strong, with a value of –25 dB, and in the time domain, it was possible to observe the modulated acoustic signal, in the case of the polarization system, the signal was weak, and the peak intensity at 130 Hz was approximately –76 dB. In addition to the main frequency, we can see other sub-harmonic frequencies in the case of interferometry system.

**Figure 9:**
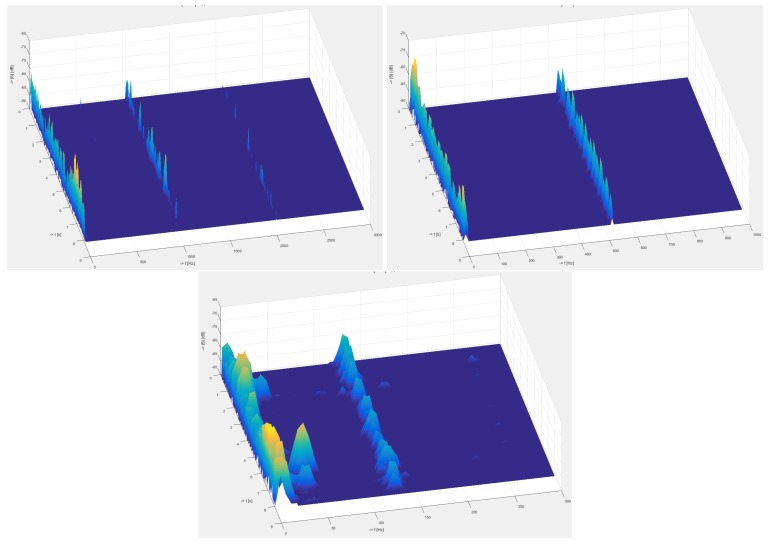
Polarization system. Comparison of calculated 3D spectrogram for all measured frequencies. (**Top-left**): 1030 Hz, (**top-right**) 530 Hz, and (**bottom**): 130 Hz.

Below, in Figure 9 are depicted calculated 3D spectrograms for all three frequencies measured by the polarization systems. The results show that with increasing frequency, the sensitivity of the system slightly decreases, and the signal-to-noise ratio (SNR) deteriorates.

### 4.4. Real Network Infrastructure Measurement

**Figure 10:**
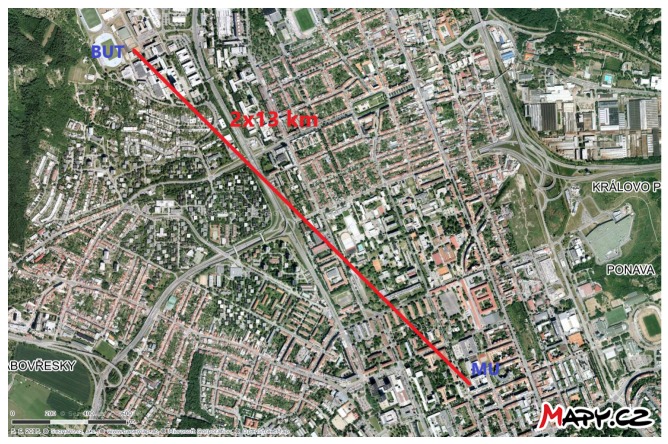
Experimental university network between Brno University of Technology and Masaryk University.

In this part, we briefly show the impact of mechanical vibrations on both systems. Compared to laboratory measurements, for polarization analysis, we used a Thorlabs polarimeter to show results on the Poincaré sphere. For this purpose, a 13 km length optical route between Brno University of Technology (BUT) and Masaryk University (MU) in the city of Brno was chosen (see Figure 10).

**Figure 11:**
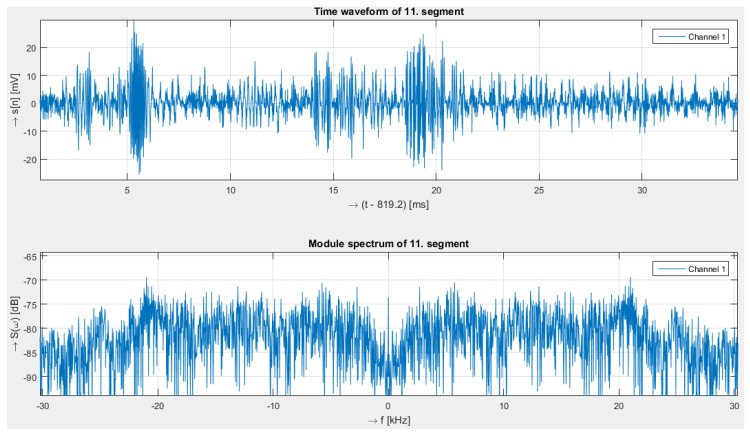
Knocking on a wall near a rack unit close to an optical fibre. Response in time (**upper**)and corresponding spectra (**below**) measured with the interferometry system.

**Figure 12:**
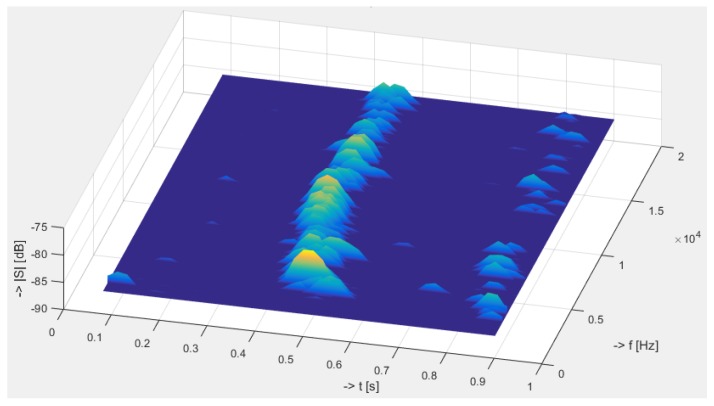
3D spectrogram. Knocking on a wall near a rack unit close to an optical fibre; measured with the interferometry system.

Figure 11 and Figure 12 show the time domain signal and its corresponding spectrum, respectively, for the mechanical vibration caused by knocking on a wall near a rack unit.

Compared to laboratory measurements, the signal was noisier. The main reason for noise was that the cable was located in the city of Brno, passing under roads or tram tracks several times. Even though, for example, tram ride passage is a relatively strong source of vibration, it was not possible to analyze these events even after postprocessing. Nevertheless, all these sources caused a certain increased noise level in the interferometric system.

**Figure 13:**
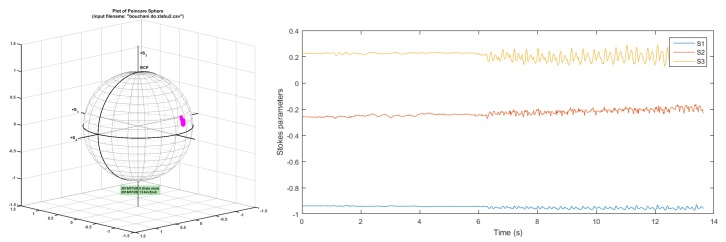
State of polarization (SOP) change caused by mechanical vibration near the cable. **L**: the Poincaré sphere, **R**: Stokes parameters.

In Figure 13 is depicted state of polarization (SOP) change caused by mechanical vibrations: knocking near the fibre optic cable. The state of polarization change is plotted on the Poincaré sphere (Figure 13 left) and is also described by Stokes parameters (Figure 13 right) that characterize the polarization change.

## 5. Conclusions

In this paper, we briefly introduced two different acoustic vibration detection techniques for fibre infrastructure protection, where both systems are capable of parallel operation with data transmission. From our results, it is obvious that the interferometry-based sensing system is much more sensitive to surrounding events than is the polarization based sensing system. Many other sub-harmonic frequencies could be caused by the use of the loudspeaker. The signal intensity from the polarization-based sensing system was low, and without post-processing, it was not possible to evaluate the events. Based on results from laboratory measurements, long-term (on the order of days) measurements on a real telecommunication network will be performed. In a subsequent work, we would like to focus on the analysis of various laser sources and their suitability for use in polarization sensing systems.
